# ERP Indices of Stimulus Prediction in Letter Sequences

**DOI:** 10.3390/brainsci4040509

**Published:** 2014-10-23

**Authors:** Edith Kaan, Evan Carlisle

**Affiliations:** Department of Linguistics, University of Florida, Box 115454, Gainesville, FL 32611, USA; E-Mail: e.carlisle1017@gmail.com

**Keywords:** predictive processing, anticipatory processing, P2, P3b, stimulus preceding negativity, SPN, omission error, MMN, contingent negative variation, CNV

## Abstract

Given the current focus on anticipation in perception, action and cognition, including language processing, there is a need for a method to tap into predictive processing in situations in which cue and feedback stimuli are not explicitly marked as such. To this aim, event related potentials (ERPs) were obtained while participants viewed alphabetic letter sequences (“A”, “B”, “C”, “D”, “E”, …), in which the letters were highly predictable, and random sequences (“S”, “B”, “A”, “I”, “F”, “M”, …), without feedback. Occasionally, the presentation of a letter in a sequence was delayed by 300 ms. During this delay period, an increased negativity was observed for predictive *versus* random sequences. In addition, the early positivity following the delay was larger for predictive compared with random sequences. These results suggest that expectation-sensitive ERP modulations can be elicited in anticipation of stimuli that are not explicit targets, rewards, feedback or instructions, and that a delay can strengthen the prediction for a particular stimulus. Applications to language processing will be discussed.

## 1. Introduction

The idea that cognition and perception are anticipatory in nature has recently regained scholarly attention [1,2]. Humans continuously make predictions concerning upcoming events or stimuli and match these predictions to the actual input. When expectations are not met, the resulting error signal is used to extract important information and adjust expectations. The ability to predict therefore underlies the ability to learn and adapt. Predictive processing is an essential part of perception, action and cognition [1,2], including language processing [3,4,5,6,7,8,9,10]. Therefore, a growing need exists for experimental techniques and designs that can tap into predictive processing. Event-related-potentials (ERPs) are an appropriate method, since this technique enables the continuous recording of brain activation during cognitive processing.

One class of ERP components associated with anticipatory processing are stimulus preceding negativities (SPNs) [11]. SPNs are a collection of negativities whose scalp distribution varies depending on the task and type of information that is expected [12,13]. SPNs are typically elicited in delay paradigms. In these paradigms, a particular stimulus is temporally separated from a subsequent stimulus, which can occur with a varying degree of predictability. SPNs have typically been observed in the period: before a stimulus that conveys feedback on the performance of the preceding stimulus; before a stimulus that conveys instructions; before a probe to which previous stimuli or task outcomes need to be compared; or before a stimulus that is expected to be associated with a strong emotion [13]. The SPN is not an index of expectancy of stimuli in general, however. First, not all stimuli that are highly predictable give rise to an SPN. For instance, the expected occurrence of an instruction stimulus at a particular point in a trial does not [14]. Second, the SPN can be larger when the nature of the following stimulus is less, rather than more, predictable [15,16,17]. One interpretation is that the SPN is larger when the participant expects the upcoming stimulus to be more emotionally informative [16,18].

Another ERP response associated with anticipation is an omission-related negativity, also regarded as a mismatch negativity (MMN) and/or an N2 component [19,20,21]. This component has been observed in auditory paradigms in which an expected stimulus is occasionally either briefly delayed or omitted and replaced with a brief period of silence. The omission-related negativity can be modulated by the degree of expectation: Its amplitude is larger when the omitted stimulus is more strongly expected [22,23]. Omission-related effects have also been reported in studies using visual presentation in which the temporal regularity of stimulus presentation is occasionally disrupted [24,25]; to our knowledge, however, no omission studies have manipulated the predictability of the upcoming stimulus in a visual paradigm.

The present study aimed to test whether anticipation-related effects could be elicited in a delay paradigm in which pre-learned visual sequences were used and no explicit feedback was provided. Participants were presented with letter sequences, which could be either in alphabetical order, in which the next letter was 100% predictable, or in a random order, in which the next letter was about 5% predictable. In both types of sequence, half of the trials had a lengthened inter stimulus interval (ISI) between two of the letters, creating a delay. The task was an end-of-trial letter recognition task, which was unrelated to the predictability of the sequence or the presence of a delay. If participants form predictions of the next letter on the basis of the preceding letters, the delay would either strengthen the expectation of the next letter or, alternatively, violate the expectation of the presentation of a particular letter at a certain point in time. In either case, we expected differences in amplitude between the predictable and random sequences.

In addition, we were interested in the effect of predictability and delay on the degree to which the next letter was pre-activated. One interpretation of anticipatory processing is that the perception of upcoming stimuli is facilitated by the pre-activation of the relevant sensory and cognitive systems. This facilitation may be reflected in the modulation of early sensory ERP components when the expected stimulus occurs. The P2 is a component typically occurring between 100 and 300 ms. The P2 has been associated with early perceptual processes, which can be modulated by top-down information. In the visual domain, a larger P2 amplitude has been associated with the ease in which relevant (pre-specified, target-related) visual features, such as a particular color or size, can be detected [26]. In the language domain, the P2 is larger for repeated words [27,28] and larger for plausible words in a highly-constraining *versus* a weakly-constraining semantic context [29,30]. Evans and Federmeier [27] (p. 305) suggest that, since the P2 is sensitive to word repetition, “it may reflect the process of comparing visual input with either stored knowledge or generated expectations”. In addition, the P300, and in particular the P3b, has been found to be larger for stimuli that are highly expected in a context and has been interpreted as indexing the matching of expectation, that is, the match of the actual stimulus with a pre-activated item [31,32,33].

We therefore expected a larger P2 and/or P3b for elements in predictive *versus* non-predictive sequences. If a lengthened ISI makes the prediction stronger and leads to more pre-activation of the anticipated element, we expected a larger P2/P3b after a delay than without a delay, especially for predictive sequences.

## 2. Methods

### 2.1. Participants

Nineteen native speakers of American English were recruited from the University of Florida community. Data from one participant were excluded because of chance performance on the end-of-trial probe verification task, leaving a total of 18 participants (age 18–30, mean 20.9; 4 men). All, except for one, participants were right-handed. All had normal or corrected-to-normal vision, had no reading problems and were neurologically healthy according to a self-report. All participants were able to generate the letters of the Roman alphabet in the correct order when asked before the study. Participants were either paid $10.00 per hour or received course credit for their participation. All participants gave written informed consent as per the University of Florida Institutional Review Board procedures (Protocol #2013-U-0768, approved 6/19/2013).

### 2.2. Materials

Stimuli consisted of 180 sequences of upper-case letters of the Roman alphabet. First, 45 predictive, alphabetical sequences (e.g., “E”, “F”, “G”, “H”, “I”, “J”, “K”) were formed. Sequences were between 7 and 10 letters long. Random sequences were derived from the predictive sequences, such that the random and predictive sequences did not differ in the number and distribution of particular letters over the positions in the sequence. When creating random sequences, strings of letters were avoided that may form known acronyms or other recognizable patterns (e.g., “OMG”, “LOL”, *etc.*). From these 90 no-delay sequences, 90 delay trials were created by adding a delay (that is, a lengthened ISI) after the fourth to eighth letter (nine instances for each of these five positions for the predictive and random conditions). Care was taken that the position of the delay in the predictive sequences did not occur in a place where a pause may feel natural due to the pauses in the “ABC Song” (after “G”, “P”, “S”, “V”, and “X”) and that the delay was never in a pre-final position. This was to prevent participants from expecting a delay in a certain position, and to encourage them to keep processing the stimuli even after the delay. Each participant saw all 180 trials (45 for each of the 4 conditions: predictive delay; random delay; predictive no delay; random no delay). Each of the trials was followed by a letter prompt. In half of the trials, equally distributed across conditions, the prompt letter was a member of the sequence (“yes” trials); in the other half, it was not (“no” trials). Prompt letters in the “yes” trials were equally drawn from the various letter positions, with the position balanced equally over the random and predictive sequences.

### 2.3. Procedure

Participants were seated comfortably in a sound-attenuating room, 1 m away from an SVGA monitor. Letters were presented in Courier New 24 point font, white letters on a black background. Each sequence began with a fixation presented in the center of the screen for 1000 ms, followed by a blank screen of 200 ms. Letters were presented individually for 300 ms followed by a blank screen (ISI) of 200 ms. In the conditions containing a delay, one of the letters in Positions 5 through 9 was followed by an ISI of 500 ms rather than 200 ms, thus creating a delay of 300 ms relative to the expected onset time. The final letter of each sequence was presented with a period. After a blank screen of 1000 ms, a prompt letter was presented. At this point, participants indicated whether or not that letter had occurred in the preceding sequence by pressing the left (“yes”) or right (“no”) trigger button on a game pad. After responding to the prompt, the message “Press to continue” remained on the screen until the participant pressed a button on the gamepad to proceed to the next trial.

Participants were instructed to limit movements and blinking to the pauses at the end of each sequence (during the prompt or after). Upon the completion of a practice block, which consisted of six sequences (three alphabetic, three random, none containing a disruption), participants completed three blocks, each consisting of 60 sequences. Each block contained 15–16 trials from each of the four conditions, in a randomized order. The order of the three blocks was varied such that no more than two participants saw the blocks in a given order.

### 2.4. EEG Recording

EEG was recorded with 64 Ag/AgCl electrodes mounted in an elastic cap (Waveguard 64). The following electrode positions were used: on the midline (FPz, Fz, FCz, Cz, CPz, Pz, POz, Pz) and lateral sites (FP1/2, AF3/4/7/8, F1-8, FC1-6, FT7/8, C1-6, T7/8, CP1-6, TP7/8, P1-8, PO3-8, O1/2). Horizontal EOG was recorded with two Ag/AgCl electrodes attached to the left and right outer canthi of both eyes, and vertical EOG was recorded with Ag/AgCl electrodes above and below the right eye. Additional electrodes were placed on the left and right mastoids. Impedances never exceeded 5 kΩ at all times for all electrodes. EEG was acquired using an ANT REFA 8-72 amplifier, at a sampling rate of 512 Hz, referenced to the left mastoid.

### 2.5. Analysis

Data were filtered offline using a 0.01–30 Hz band pass filter and arithmetically re-referenced to the mean of both mastoids. Trials with excessive eye movements (standard deviation exceeding 20 µV for horizontal EOG; greater than 30 for vertical EOG in a 200-ms window) and trials in which one of the channels was malfunctioning as determined by visual inspection were rejected from analysis. The rejection rate was equally distributed among the four conditions (predictive, delay 16.5%; predictive, no delay, 16.5%; random, delay 16.4%; random, no delay 16.2%). ERPs were time-locked to the onset of the presentation of the letter preceding the potential delay. Epochs spanned 2000 ms, from −500 to 1500 ms, relative to stimulus onset. As will be explained below, the 200-ms time window immediately preceding the pre-delay letter may have been confounded by pre-existing differences between the conditions. For this reason, we report an analysis using the −500 to −300-ms interval preceding the onset of the pre-delay letter as the baseline. This window coincided with the first 200 ms after onset of the second letter preceding the potential delay. To investigate the effects of random *versus* predictive sequences, without contamination of the nature or the preceding stimuli or presence of a delay, we also investigated the ERP for the first three letters in the trial, using the 100-ms interval preceding the onset as a baseline (a longer baseline was too contaminated with eye movements spilling over from the preceding fixation). Epochs were 1600 ms in length, from −100 to 1500 ms from the onset of the first letter.

Statistical analyses were performed on the mean of five of the midline electrodes (Fz, FCz, Cz, CPz, Pz) and 30 lateral electrodes grouped into 10 regions (left frontal: F1/3/5; right frontal: F2/4/6; left fronto-central: FC1/3/5; right fronto-central: FC2/4/6; left central: C2/4/6; right central: C1/3/5; left centro-parietal: CP2/4/6; right centro-parietal: CP1/3/5; left parietal: P2/4/6; and right parietal: P1/3/5). Critical time windows were: (1) −300 to 0 ms, 0–300 ms and 300–500 ms relative to the onset of the pre-delay letter; this was to examine potential differences between the conditions before the onset of the delay; (2) 600 to 900 ms after onset of the pre-delay letter and corresponding letter in the no-delay conditions; this interval captured the delay-related ERPs; (3) the P2/P3b, which was defined as the 100-ms window surrounding the peak latency averaged over midline sites. This corresponded to the 680–780-ms window from the start of the pre-delay letter for the no-delay conditions and the 980–1080-ms window for the delay conditions. The critical time windows for the analysis of the first three letters of each trial were: (1) 690–780-ms window from the onset of the first letter, capturing the P2/P3 to the second letter; (2) the mean amplitude between 800 and 1000 ms; and (3) 1300–1500 ms after the onset of the first letter, based on visual inspection. SPSS GLM repeated measures analyses were performed on the mean amplitude in the time windows defined, separately for midline and lateral sites. Within-subject factors were order (2 levels: predictive/random), delay (2 levels: delay/no delay), when applicable, and anteriority (5 levels: frontal, fronto-central, central, centro-parietal, parietal). Lateral analyses were performed with the addition of hemisphere (2 levels: left/right) as a factor. Significant interactions involving the factors delay or order were followed up with ANOVAs on the relevant subset of the data. For effects involving more than one degree of freedom, the Greenhouse–Geisser correction was applied to protect against Type I error due to a violation of sphericity [34].

Accuracy for the end-of-trial probe verification task and response times to accurate responses were analyzed with an SPSS GLM repeated measures with order (2) and delay (2) as within-subjects factors. Outliers in reaction times were omitted when they exceeded the median plus 3-times the median absolute deviation [35]. This affected 8.3% of all data points.

## 3. Results and Discussion

### 3.1. Behavioral Data

The mean proportion of accurate responses and response times for accurate responses in the end-of-trial letter recognition task are given in Table 1. Participants were very accurate in indicating whether the post-trial prompt letter was among the letters seen in the trial. Responses were more accurate, but took longer, in the predictive than in the random conditions (accuracy, *F* (1, 17) = 23.85, *p* < 0.001; response times, *F* (1, 17) = 23.46, *p* < 0.001), suggesting a speed/accuracy trade-off. Responses were more accurate (*F* (1, 17) = 4.81, *p* < 0.05) and faster (*F* (1, 17) = 11.16, *p* < 0.05) when the sequence did not contain a delay, suggesting that the presence of a delay hindered the encoding or retrieval of the sequence.

**Table 1:** Mean proportion of accurate responses and mean response times (RT) in ms to accurate responses in the letter recognition task (standard deviation in parentheses).

Condition	Mean Accuracy (SD)	Mean RT (SD)
Predictive, delay	0.94 (0.04)	1225 (231)
Random, delay	0.88 (0.06)	1153 (209)
Predictive, no delay	0.96 (0.04)	1195 (232)
Random, no delay	0.89 (0.06)	1099 (216)

### 3.2. ERPs: Delay-Related Effects (Time Locked to Pre-Delay Letter)

**Figure 1:**
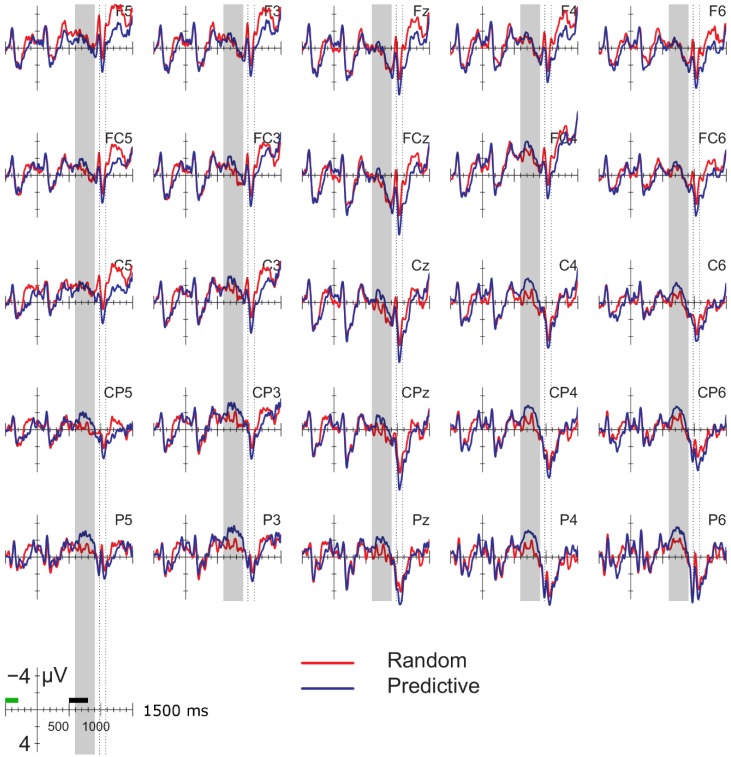
Event-related potentials (ERPs) time-locked to the onset of the pre-delay letter in the delay conditions for 25 selected electrodes (left hemisphere (F3, F5, FC3, FC5, C3, C5, CP3, CP5, P3, P5); midline (Fz, FCz, Cz, CPz, Pz); right hemisphere (F4, F6, FC4, FC6, C4, C6, CP4, CP6, P4, P6). The delay period (500–800 ms) is indicated with the black bar above the time scale; the letter following the delay starts at 800 ms. Blue line: predictive (alphabetic) condition; red line: random condition. Gray vertical bar: 600–900-ms interval used for the analysis of the effects during the delay; vertical dotted lines: analysis window for the P2/P3 effects. ERPs are baselined to the 200 ms following the onset of the second letter before the potential delay (−500 to −300 ms, see the green bar above the time scale). In this and the following figures, negative is plotted up.

**Figure 2:**
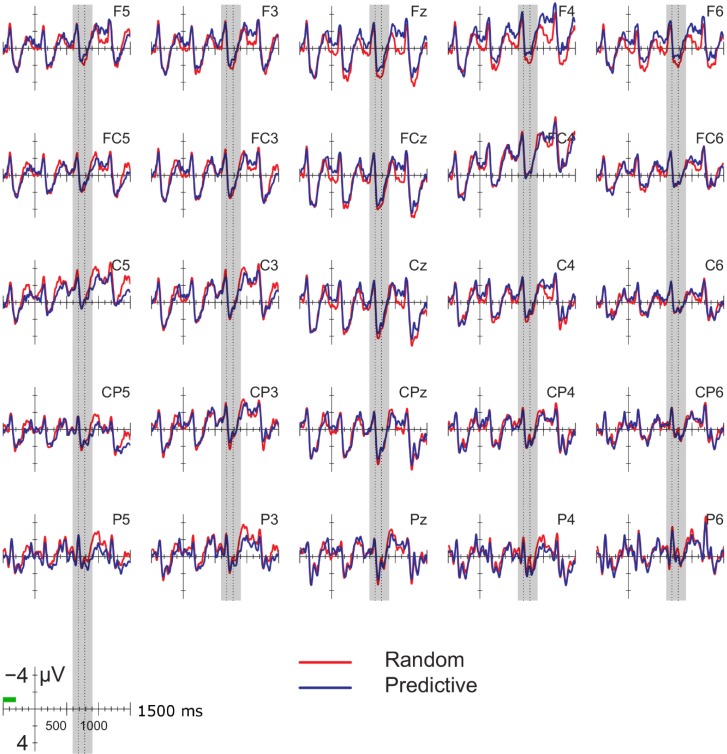
ERPs for the no-delay conditions, time-locked to the onset of the letter equivalent in position to the pre-delay letter in Figure 1. The next letter starts at 500 ms. Blue line: predictive (alphabetic) condition; red line: random condition. ERPs are baselined to the −500 to −300 ms interval (green bar). Gray vertical bar: 600–900 ms interval used in the analysis of the delay effects; vertical dotted lines: analysis window for the P2/P3 effects.

Figure 1 displays the ERPs time-locked to the onset of the pre-delay letter in the delay conditions; Figure 2 displays the ERPs for the corresponding position in the no-delay conditions. The delay occurred 500–800 ms after the onset of the pre-delay letter. Shortly after the onset of the delay, a negative going potential was seen (600–900 ms, gray vertical bars in Figure 1), which was larger for the predictive than the random sequences. In addition, letters following the delay elicited a larger positivity between 180 and 280 ms after onset than letters in comparable positions without a delay (dotted interval in Figure 1 and Figure 2). This positivity was larger in amplitude for the predictive than for the random sequences, but only after a delay. Superimposed on this effect was a frontal negativity for the random *versus* the predictive delay conditions, which lasted until the end of the epoch. Note that the random and predictive conditions differed right before the onset of the pre-delay letter, −300 to 0 ms in Figure 1 and Figure 2. In this time-window, the ERPs in the random conditions were more negative than in the predictive conditions, which is the reverse pattern of that observed during the delay period.

#### 3.2.1. Pre-Delay Period

In order to assess potential differences in ERPs between the conditions before the delay, we conducted analyses on the 300-ms time window preceding the pre-delay letter, and on the 0–300-ms and 300–500-ms time windows from the onset of the pre-delay letter.

In the 300-ms interval preceding the pre-delay letter, the ERPs in the random conditions were more negative than in the predictive conditions at lateral sites, which just failed to reach significance (*F* (1, 17) = 4.352, *p* = 0.052). This is the opposite pattern of that found in the delay period, where the ERPs to the predictive condition were more negative than the random. No other significant effects were obtained in the −300 to 0-ms or 0–300-ms intervals. In the 300–500-ms time window, no differences were seen among the conditions except for a significant interaction of delay, anteriority and hemisphere at lateral sites (*F* (4, 68) = 5.635, *p* < 0.01). This effect could be attributed to the ERPs being more negative in the no-delay than delay conditions over left posterior sites. The direction of this delay effect is opposite of that found in the analysis of the delay interval, during which the ERPs in the delay conditions were more negative. The differences observed in later intervals therefore cannot be ascribed to differences seen before the delay.

#### 3.2.2. Delay Period

ERPs shortly after the onset of the delay period (see Figure 1) were more negative over posterior sites compared to when there was no delay (Figure 2). This delay-related negativity was larger for the predictive than the random sequences, whereas there were no differences between the random and predictive order in the no-delay conditions. These observations were confirmed by statistical analyses of the mean in the 600–900-ms interval after the onset of the pre-delay letter. Results from the ANOVA are presented in Table 2. Analyses showed a robust interaction of delay and anteriority. Separate analyses for each of the anterior-posterior sites (Table 3) confirmed that the delay-related negativity was most prominent at posterior sites.

**Table 2:** Results from ANOVA (*F*-values) on the mean amplitudes in the 600–900-ms interval from the onset of the pre-delay letter.

Effect (df)	Midline	Lateral
Order (1,17)	2.037	1.531
Delay (1,17)	4.094 ^+^	11.678 **
Order × Anteriority (4,68)	0.246	0.155
Delay × Anteriority (4,68)	6.717 **	9.566 **
Order × Hemi (1,17)	–	0.564
Delay × Hemi (1,17)	–	0.820
Order × Anteriority × Hemi (4,68)	–	1.317
Delay × Anteriority × Hemi (4,68)	–	3.451 *
Order × Delay (1,17)	0.951	4.154 ^+^
Order × Delay × Anteriority (4,68)	2.052	3.418 ^+^
Order × Delay × Hemi (1,17)	–	0.009
Order × Delay × Anteriority × Hemi (4,68)	–	0.170
Anteriority (4,68)	4.513 **	0.878
Hemi (1,17)	–	0.000
Anteriority × Hemi (4,68)	–	1.219

df: degrees of freedom; Hemi: hemisphere; ^+^ *p* < 0.1; * *p* < 0.05; ** *p* < 0.01.

**Table 3:** Follow-up analysis of the delay × anteriority effect, 600–900 ms from the onset of the pre-delay letter. *F*-values for the effect of delay are given for each anteriority region.

Region	Midline, F	Lateral, F
Frontal	0.043	0.365
Fronto-Central	0.011	1.520
Central	3.033	7.566 *
Central-Parietal	11.731 **	15.640 **
Parietal	14.081 **	21.994 ***

df: degrees of freedom; * *p* < 0.05; ** *p* < 0.01; *** *p* < 0.001.

**Figure 3:**
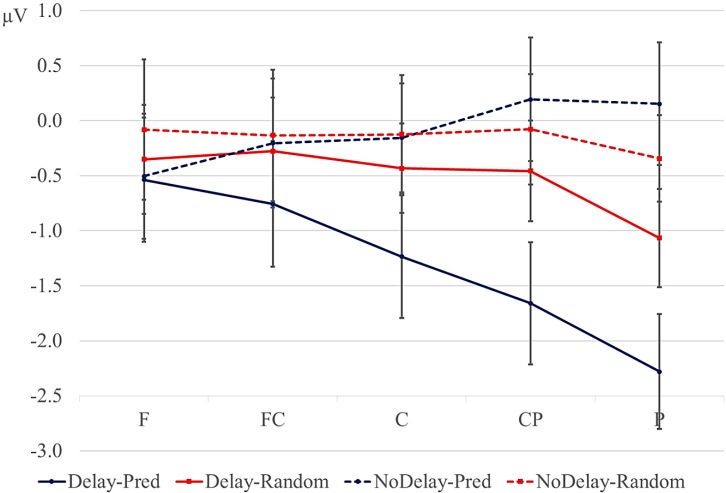
Mean amplitude (µV) in the 600–900-ms window for the lateral regions, collapsed over hemisphere (error bars are standard errors). Blue lines: predictive sequences (Pred); red lines: random sequences; solid lines: delay conditions; dotted lines: conditions without a delay. F: frontal; FC: fronto-central; C: central; CP: central-parietal; P: parietal.

The overall analyses showed a marginal interaction between order and delay at lateral sites (order × delay, *F* (1, 17) = 4.154, *p* = 0.06; order × delay × anteriority *F* (4, 68) = 3.418, *p* = 0.08). Since we had specifically predicted differences between the random and predictive conditions as a function of the delay, we conducted separate analyses for the delay and no-delay conditions at lateral sites. Results are reported in Table 4. The predictive sequences were more negative than random sequences only in the delay conditions. Results of the follow-up analyses of the effects of order of each anteriority region within the delay condition are given in Table 5. These confirm the observation that the negativity for predictive *versus* random condition is largest at posterior sites, bilaterally. Means for the lateral sites are given in Figure 3.

**Table 4:** Delay *versus* no-delay, 600–900 ms; results from ANOVA (F-values) for lateral sites.

Effect (df)	Delay	No Delay
Order (1,17)	4.908 *	0.018
Order × Anteriority (4,68)	5.502 *	0.920
Order × Hemi (1,17)	0.111	0.223
Order × Anteriority × Hemi (4,68)	0.884	0.602
Anteriority (4,68)	3.737 **	0.273
Hemi (1,17)	0.098	0.094
Anteriority × Hemi (4,68)	0.609	2.118

df: degrees of freedom; Hemi: hemisphere; * *p* < 0.05; ** *p* < 0.01.

**Table 5:** Delay conditions only. Follow up analysis of the order × anteriority effect, 600–900 ms from the onset of the pre-delay letter at lateral sites. *F*-values for the effect of order are given for each anteriority region.

Region	Lateral, F
Frontal	0.175
Fronto-Central	1.694
Central	5.074 *
Central-Parietal	10.978 **
Parietal	8.623 **

df: degrees of freedom; * *p* < 0.05; ** *p* < 0.01.

#### 3.2.3. Post-Delay: 180–280-ms Positivity

**Figure 4:**
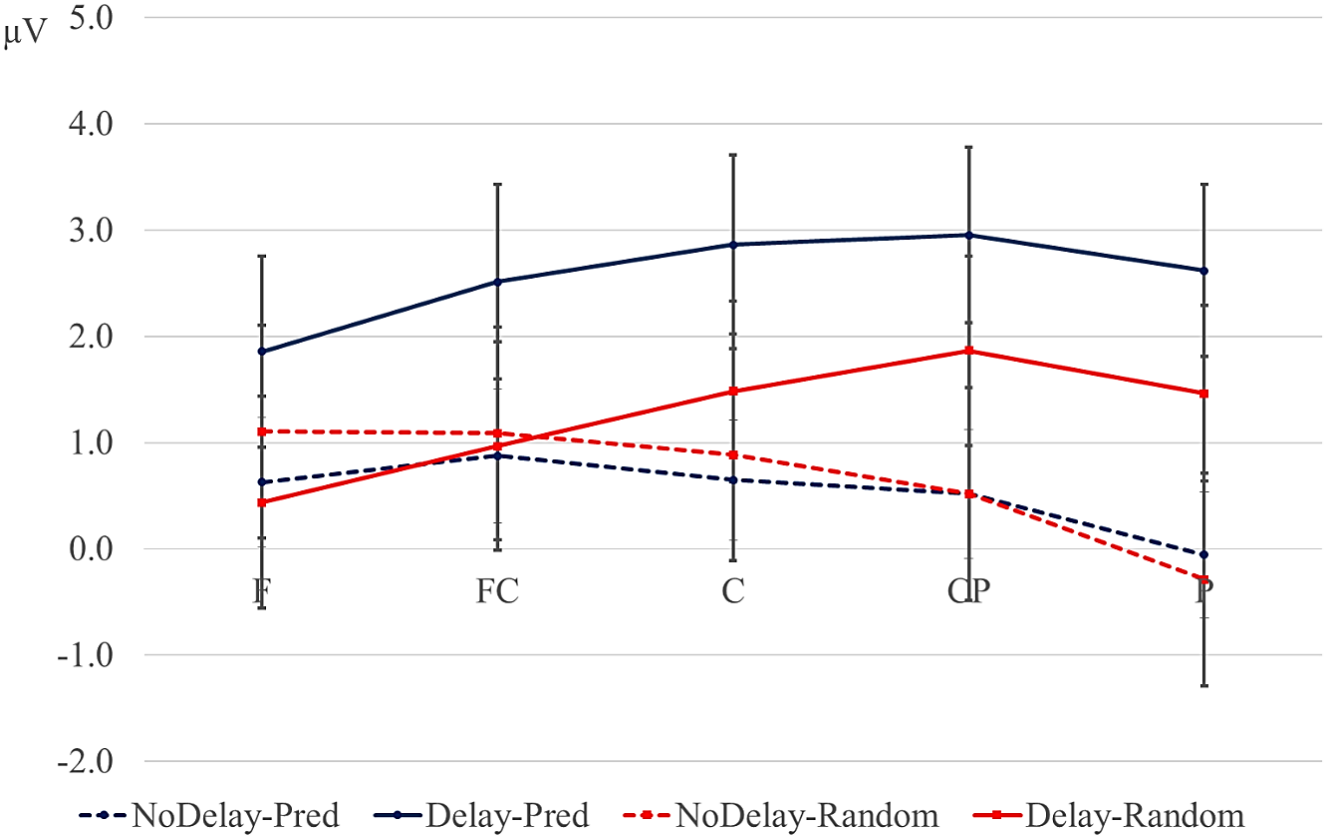
Mean amplitude (µV) between 180 and 280 ms from the onset of the post-delay letter, for the lateral positions, collapsed over hemisphere (error bars are standard errors). Blue lines: predictive sequences (Pred); red lines: random sequences; solid lines: delay conditions; dotted lines: conditions without a delay. F: frontal; FC: fronto-central; C: central; CP: central-parietal; P: parietal.

Letters directly following a delay elicited a larger positivity between 180 and 280 ms at posterior sites compared with letters in similar positions in no-delay trials (see the dotted interval in Figure 1 and Figure 2). This effect was largest over right-hemispheric sites. In addition, the positivity was larger for predictive than for random sequences, but only in the delay conditions. This order effect was broadly distributed (Figure 4). Table 6 gives an overview of the results from the statistical analyses on the mean amplitude of the positivity (that is, 680–780 ms post onset of the preceding letter for the no-delay conditions, 980–1080-ms onset of the pre-delay letter for the delay conditions). Follow-up analyses of the significant interactions between the factors, delay and anteriority, support the observation that the positivity after the delay is largest at posterior sites (Table 7). To assess the delay × hemisphere interaction, separate analyses were conducted for left and right lateral sites. The effect of delay was significant only over right hemispheric sites (right hemisphere, mean delay effect: 2.25 µV; *F* (1, 17) = 13.627, *p* < 0.01; left hemisphere, mean delay effect: 0.37 µV; *F* (1, 17) < 1).

The interaction of order and delay was followed up by analyses of the delay and no-delay conditions separately; see Table 8. The positivity was significantly larger for the predictive than the random conditions only in the delay conditions. In contrast to the delay-related negativity (Section 3.2.2), the effect of order did not interact with anteriority (see Figure 4).

**Table 6:** Results from ANOVA (*F*-values) on the mean amplitudes in the 180–280-ms interval from the onset of the post-delay letter (680–780 ms and 980–1080 ms from the preceding letter in the no delay and delay conditions, respectively).

Effect (df)	Midline	Lateral
Order (1,17)	1.974	3.069 ^+^
Delay (1,17)	3.756 ^+^	5.114 *
Order × Anteriority (4,68)	0.214	0.256
Delay × Anteriority (4,68)	8.477 **	10.299 **
Order × Hemi (1,17)	–	0.306
Delay × Hemi (1,17)	–	33.616 ***
Order × Anteriority × Hemi (4,68)	–	1.193
Delay × Anteriority × Hemi (4,68)	–	2.169
Order × Delay (1,17)	14.765 **	14.022 **
Order × Delay × Anteriority (4,68)	0.693	0.688
Order × Delay × Hemi (1,17)	–	0.054
Order × Delay × Anteriority × Hemi (4,68)	–	0.268
Anteriority (4,68)	1.717	0.703
Hemi (1,17)	–	1.865
Anteriority × Hemi (4,68)	–	1.810

df: degrees of freedom; Hemi: hemisphere; ^+^ *p* < 0.1; * *p* < 0.05; ** *p* < 0.01; *** *p* < 0.001.

**Table 7:** *F*-values for the effect of delay, for each region, 180–280 ms from the onset of the post-delay letter.

Region	Midline df (1,17)	Lateral df (1,17)
Frontal	0.244	0.206
Fronto-Central	1.102	1.417
Central	2.120	5.807 *
Central-Parietal	6.526 *	9.152 **
Parietal	12.400 **	12.075 **

df: degrees of freedom; * *p* < 0.05; ** *p* < 0.01.

**Table 8:** Results for follow-up ANOVA (*F*-values), 180–280 ms from the post-delay letter, separately for delay and no-delay conditions.

Effect (df)	Delay (980–1080 ms)		No Delay (680–780 ms)	
	Midline	Lateral	Midline	Lateral
Order (1,17)	9.032 **	10.581 **	1.242	0.141
Order × Anteriority (4, 68)	0.967	0.732	0.309	0.483
Order × Hemi (1,17)	–	0.016	–	0.302
Order × Anteriority × Hemi (4, 68)	–	0.667	–	0.919
Anteriority (4, 68)	2.318	1.515	4.029 *	3.374 ^+^
Hemi (1,17)	–	9.862 **	–	1.265
Anteriority × Hemi (4, 68)	–	2.069	–	1.435

df: degrees of freedom; ** *p* < 0.01.

#### 3.2.4. Other Effects

As can be seen in Figure 1, the 180–280-ms positivity overlapped with a longer-lasting frontal negativity for the random *versus* predictive delay conditions. This observation was supported by a significant interaction between anteriority and order for the 1200–1400 ms interval from the onset of the pre-delay letter, that is 400–600 ms from the onset of the post-delay letter (midline: *F* (4, 68) = 5.603, *p* < 0.05; lateral: *F* (4, 68) = 7.356, *p* < 0.01). Follow-up analyses confirmed that the effect of order was present only at frontal sites (frontal lateral regions: *F* (1, 17) = 4.561, *p* < 0.05; other regions, *p*s *>* 0.1). Although the effect seemed largest over left frontal and central sites, the three-way interaction between order, anteriority and hemisphere did not reach significance (*F* (4, 68) = 1.364, *p* = 0.26).

Finally, visual inspection of the results for the no-delay conditions in Figure 2 suggested that ERPs for the random sequences were more negative than for the predictive conditions at left posterior sites between 800 and 1000 ms and more positive at frontal sites between 1000 and 1200 ms after the onset of the pre-delay letter equivalent. However, analyses for these two intervals in the no-delay conditions yielded no significant effects involving the factor order (order × hemisphere 800–1,000 ms, *F* (1, 17) = 3.197; *p* = 0.09; other effects, *p*s > 0.5).

### 3.3. ERPs: Effects of Predictive *vs*. Random Sequences (Time Locked to First Letter)

**Figure 5:**
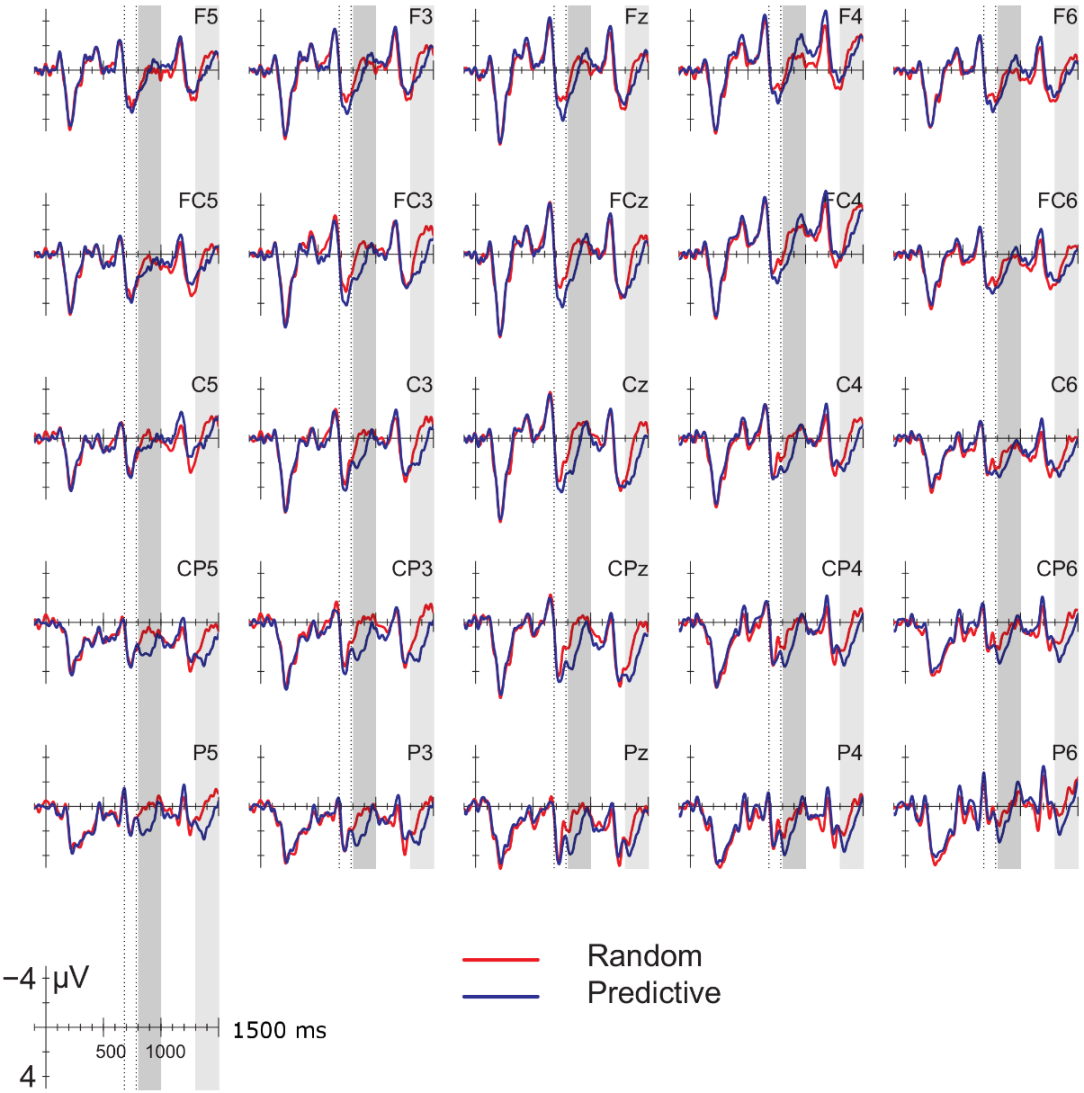
ERPs time-locked to the onset of the first letter in the sequence. ERPs are baselined to 100 ms preceding the onset of the first letter. Letters are presented for 300 ms, starting at 0 ms, 500 ms and 1000 ms. Blue line: predictive (alphabetic) condition; red line: random condition. Dotted lines: window for the analysis of the P2; shaded intervals: windows used for the analysis of the negativity (dark bar: 800–1000 ms; light bar: 1300–1500 ms).

**Figure 6:**
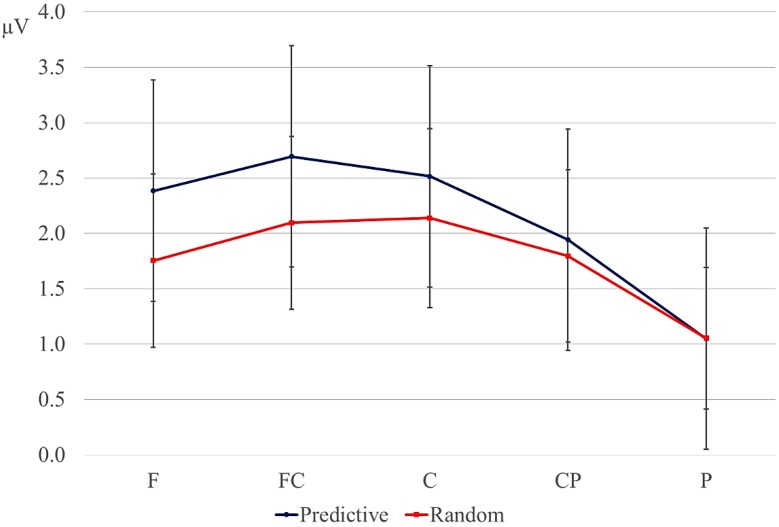
Mean amplitude (µV) between 180 and 280 ms from the onset of the second letter in the trial, for the lateral positions, collapsed over hemisphere and delay (error bars are standard errors). Blue lines: predictive sequences; red lines: random sequences. F: frontal; FC: fronto-central; C: central; CP: central-parietal; P: parietal.

In order to further explore differences between random and predictive sequences, we investigated ERPs to the first few letters of each trial. At the first letter of each trial, participants did not know whether the sequence was random or predictive. Only the second letter was informative in this respect. Differences in ERPs at the second letter were therefore not confounded by pre-existing differences between the predictive and random conditions. Figure 5 displays the ERPs starting from the presentation of the first letter in the sequence. The P2 to the second letter was larger for the predictive than for the random conditions, especially at anterior sites (Figure 6). Results from the ANOVA are given in Table 9. In the window spanning the P2 at the second letter (680–780 ms from the onset of the first letter), a marginal interaction between order and anteriority was found in the analysis of the lateral sites (*p* = 0.051). Follow-up analyses for each anteriority region (Table 10) supported the observation that the increased positivity for predictive *versus* random sequences was largest at frontal and fronto-central sites.

**Table 9:** First letter, results from ANOVA (*F*-values) for the 680–780-ms (P2), 800–1000-ms and 1300–1500-ms (negativity) intervals.

Effect (df)	680–780 ms		800–1000 ms		1300–1500 ms	
	Midline	Lateral	Midline	Lateral	Midline	Lateral
Order (1,17)	5.745 *	1.703	10.330 **	6.733 *	6.300 *	6.246 *
Order × Anteriority (4,68)	1.174	3.685 ^+^	4.058 *	5.890 **	1.953	2.598
Order × Hemi (1,17)	–	0.017	–	1.835	–	0.338
Order × Anteriority × Hemi (4,68)	–	0.409	–	1.577	–	0.637
Anteriority (4,68)	1.028	4.793 *	3.142 *	1.961	2.916 ^+^	2.543 ^+^
Hemi (1,17)	–	4.621 *	–	0.686	–	0.054
Anteriority × Hemi (4,68)	–	0.303	–	0.675	–	0.518

df: degrees of freedom; Hemi: hemisphere; ^+^ *p* < 0.1; * *p* < 0.05; ** *p* < 0.01.

**Table 10:** First letter, *F*-values for the effect of order, for each anteriority region, 680–780 ms (P2) and 800–1000 ms (negativity).

Region	680–780 ms	800–1000 ms	
	Lateral, F	Midline, T	Lateral, F
Frontal	3.831 ^+^	0.866	0.079
Fronto-Central	4.947 *	1.961 ^+^	3.676 ^+^
Central	1.887	3.205 **	6.459 *
Central-Parietal	0.212	3.942 **	9.414 **
Parietal	0.000	3.626 **	11.055 **

df: degrees of freedom; ^+^ *p* < 0.1; * *p* < 0.05; ** *p* < 0.01.

Second, resembling the pattern observed for the interval preceding the pre-delay letter (Section 3.2.1), the random and predictive conditions differed in that the ERPs at posterior sites were more negative for the random than for the predictive condition between the offset of the second letter (800 ms) and the onset of the next letter (1000 ms). This negativity was repeated, although less robustly, at the next letter in the sequence (1300–1500 ms). Results from the ANOVA are given in Table 9. In the 800–1000-ms window, a significant interaction was found between order and anteriority. Separate analyses for each of the anteriority regions (Table 10) confirmed that the negativity for random *versus* predictive sequences had a posterior maximum. Between 1300 and 1500 ms, only the effect of order was significant.

## 4. Discussion

The aim of the present study was to probe implicit predictive processes during the processing of alphabetical *versus* random letter sequences using a delay paradigm without feedback. We occasionally lengthened the ISI between two letters by 300 ms. This manipulation allowed us to observe whether: (1) the ERPs elicited during this delay period were sensitive to the difference in predictability of the upcoming stimulus; and (2) the delay and predictability were affected the pre-activation and processing of the next letter. During the delay period, the predictive sequences elicited a larger posterior negativity than the random sequences. In contrast, random *versus* predictive conditions showed a 300–500-ms negativity in some positions without or before a delay. Second, letters following the delay showed a larger posterior positivity between 180 and 280 ms after the onset of the post-delay letter than letters in comparable positions in the no-delay sequences. The 180–280-ms positivity was larger for predictive than for random letters following a delay. No effect of predictability on the positivity was seen in the no-delay comparisons, although we did observe such a modulation at the second letter from the beginning of each trial. We will discuss these effects in turn.

### 4.1. Delay-Related Negativity

The larger negativity observed during the delay for the predictive *versus* random sequences can be interpreted in several ways. First, it can be an SPN, indexing the expectation of the upcoming letter; second, the increased negativity can reflect a violation of the expected presentation of the next letter; third, the negativity may be related to task-effects. We will discuss these interpretations in turn.

SPNs have typically been observed when the participant expects a stimulus that is informative, either because it is a response probe, instructional probe, feedback, a reward or an affective stimulus [13]. In our study, the letter stimuli in the trials are not informative in the sense of being instructions, probes or feedback, but we do find a larger negativity in the interval preceding highly predictive letters compared with random letters. A larger SPN for predictive sequences is at odds with previous observations that the SPN is smaller preceding highly predictable compared with less predictable stimuli [15,16,17], but see [18]. Even though the letters in our alphabetical sequences were 100% predictable and, strictly speaking, uninformative, participants could have engaged themselves by internally generating the next letter during the delay interval in the predictive trials; this as opposed to the random sequences, in which a correct prediction of the upcoming letter was not very likely. The next letter in the predictive sequences then served as implicit feedback that the participant used to check the internally generated prediction. The SPN then reflects the expectation of this implicit feedback stimulus. The support for this interpretation is that the 180–280-ms positivity component after the delay was larger for the predicted than for the random letters (see below). This suggests that the form and/or concept of the upcoming letter was anticipated and pre-activated during the delay period. A potential problem with interpreting the delay-related negativity as a reflection of the anticipation of the identity of the next letter is that no such negativity was found in the no-delay predictive conditions, even though participants would be likely to pre-activate the next letter in the no-delay conditions, as well. Instead, the ERPs in the random no-delay conditions were more negative at posterior sites relative to the predictive no-delay conditions, especially at earlier positions in the trial. Given its scalp distribution and timing, we speculatively interpret the posterior negativity for the random *versus* predictive conditions as an N400 effect. Since the letters in the predictive, alphabetic sequences were highly associated, the larger N400 effect may index weaker priming between the letters in the random sequences. If the negativity we observed for the predictive sequences during the delay period is an SPN, one must therefore assume that the expectation of the particular identity of the next letter is boosted by the delay. Without the delay, the expectation is not strong enough to overcome priming-related effects.

An alternative interpretation of the delay-related negativity is that it reflects an omission error [19,20,21,36]. Given the fixed presentation rate of the stimuli preceding the delay in our study, the participant may have anticipated the presentation of the next letter at a fixed point in time. This expectation is violated by the delay, giving rise to the negativity. Assuming that the expectation for the presentation of the following letter was stronger in the predictive than random conditions, the increase in negativity in the former condition can be accounted for [22,23]. The posterior distribution of the negativity matches the posterior effects found in some omission-studies using visual stimuli [24,25]. Somewhat problematic for interpreting the negativity in terms of an omission error is that 50% of the trials in the current study contained a delay after the fifth or later letter in the sequence. The delay could therefore have been anticipated to some extent by the participants, making it somewhat unlikely that the negativity reflects an omission error. Furthermore, one would expect both the random and predictive conditions to elicit an omission-related negativity, as in both cases, the presentation of the letters is rhythmic and the delay violates this temporal regularity. Although a better control condition is needed to evaluate this prediction, the ERPs to the random delay condition seem to hardly deviate from the baseline (Figure 2 and Figure 3).

Other interpretations of the delay-related difference between random and predictive sequences cannot be excluded. One confounding factor may have been the use of a letter verification task at the end of each trial, which may have led to processing differences between the random and predictive conditions. In the debriefing after the study, some participants mentioned they encoded only the first and last letters of a predictive sequence. At the probe, they would reconstruct the sequence in between the first and last letters to match the probe. In the random sequences, on the other hand, they reported encoding each letter as it appeared. Results from the letter verification task match these observations: responses to the predictive sequences were more accurate, but slower than responses to the random sequences. The difference in ERPs between the predictive and random sequences observed during the delay period may therefore also be related to a deeper encoding for the random sequences. Somewhat problematic for this interpretation, however, is that deeper encoding is associated with a larger positivity [37,38]. Although the random conditions were relatively more positive than the predictive conditions during the delay, the random conditions lack a clear positive going component.

In order to distinguish among the interpretations of the negativity, follow-up studies are needed in which the task demands, the degree of predictability of the next stimulus and the predictability of the delay are varied. If the negativity is indeed sensitive to the predictability of the next stimulus, the negativity is expected to vary as a function of predictability [15] (but see [39]), but not as a function of the predictability of the delay. If, on the other hand, the negativity is an omission error, it will not occur when a delay is predicted to occur. If the negativity we observed is due to task demands, we should no longer observe the amplitude difference between predictive and random sequences with a different task. Although more research is needed, the current finding that the delay-related negativity was sensitive to the difference in predicted *versus* random sequences supports the view that the negativity reflects differences in expectation and that similar delay-paradigms can be used to further study predictive processing.

### 4.2. 180–280-ms Positivity: P3b and P2

We observed two modulations of the 180–280-ms positivity at the letter following the potential delay. First, a larger posterior positivity was elicited after a delay compared with letters in similar positions not following a delay. Given its posterior distribution, we identify this positivity as the P3b, which is typically seen for task-related targets and is larger when the participant devotes more resources to the task [40,41]. Under this interpretation, the delay in our paradigm may have focused the participants’ attention on the upcoming stimulus letter. Alternatively, or in addition, the stronger positivity after the delay may be related to refractory effects: early visual and auditory components have been found to be larger in amplitude with longer ISIs [42,43,44,45].

Second, we found that the 180–280-ms positivity was larger for the predictive than random sequences, but only after a delay. This effect of order could be seen over the entire scalp. One interpretation is that the effect is a modulation of the P2 component. The P2 has been associated with the ease of the detection of particular sensory features [26] and with the matching of the actual visual input with an expected form [27]. Given this interpretation, our data suggests that the delay period enabled our participants to generate an expectation of the form of the next letter in the predictive sequences. This pre-activated the visual system and led to a larger P2 for the predictive *versus* non-predictive sequences. Similarly, the 180–280-ms positivity was larger for the predictive than for the random sequences at the second position in each trial, although this effect was more frontally distributed. The increased positivity for the predictive conditions may have reflected the pre-activation of the next letter in the alphabet upon seeing the first letter in the trial. The absence of the P2 effect at later (no-delay) positions suggests that the effects of expectation on visual processing are strongest when there is less adaption of the P2 to the visual stimuli. We are somewhat cautious in interpreting the difference observed between the random and predictive prediction after the delay as a P2, however, since the difference between the conditions may have been caused or enhanced by the anterior negativity for the random conditions observed after the delay. This continued anterior difference between predictive and random conditions can be interpreted as a memory related component for the random condition [46]. As mentioned previously, the random conditions may have required more encoding. This encoding may have been more taxing directly following a potentially distracting delay.

Note that even at posterior positions, where the superimposed negative effect was least apparent, the positivity following the delay was larger for the predictive than the random conditions. This suggests that also the P3b is sensitive to predictability of the stimulus [31,32,33]. This supports the view that the delay leads to a stronger pre-activation of the upcoming stimulus and, therefore, a more apparent match of expectancy when the actual stimulus appears, leading to a larger P3b. Regardless of whether the positivity is interpreted as a P2 or P3b, our results lend support to the view that an expected form or concept is pre-activated during the delay and subsequently matched against the actual stimulus.

## 5. Conclusions

Most previous studies on anticipation in sequential processing have used paradigms in which the participant learns to associate a particular stimulus with a particular outcome and in which explicit feedback is provided. We have shown that an anticipation-related negativity can be elicited by stimuli in sequences that are based on long-term associations and in which no feedback is provided. In addition, our results suggests that some features of the anticipated stimulus are pre-activated during the delay period.

The present paradigm can be applied to the study of predictive processing during sentence comprehension. In contrast to the delay paradigms used in non-language studies, most studies on anticipation in language processing have focused on ERPs elicited by words that violate or meet context-based predictions [47,48,49,50,51,52]. ERPs at these word positions also reflect integration and revision processes, which makes it hard to distinguish predictive processing from other processes. The delay paradigm can be easily applied to sentence processing [17]: the next word in a sentence can be occasionally delayed, enabling the tracking of anticipatory processes separately from integration, while still encouraging readers to process the materials following the delay, while avoiding anomalies inherent to omission [22] and violation paradigms [47,48,49,50,51,52]. We are currently applying the delay paradigm to sentence processing in which the cloze probability of the post-delay word is manipulated [53]. In addition, by manipulating the length of the delay, one can yield a better view of the time needed for certain predictions to be generated and whether there are differences in populations in this respect, such as between second-language learners with different levels of proficiency. In sum, the delay paradigm may be a fruitful method to determine the nature and time course of anticipations during the processing of sequential stimuli, be it linguistic or otherwise.
